# Impact of assigned care providers on involvement, information, and emotional support to cancer patients

**DOI:** 10.1007/s00520-025-10179-4

**Published:** 2025-12-02

**Authors:** Helena Fohlin, Helena Tufvesson Stiller, Srinivas Uppugunduri, Marcus Schmitt-Egenolf

**Affiliations:** 1 Regional Cancer Center Southeast Sweden, Linköping, Sweden; 2 Department of Biomedical and Clinical Sciences, Linköping University, Linköping, Sweden; 3 Department of Public Health and Clinical Medicine, Umeå University, Umeå, Sweden

**Keywords:** Patient-reported experience measures, Patient involvement, Information, Emotional support, Assigned care provider, Cancer

## Abstract

**Purpose:**

Patient-reported experience measures represent the patient’s voice and offer a way to evaluate continuity of care. We investigated the impact of having an assigned care provider on experienced involvement, information, and emotional support.

**Methods:**

Data from a national survey sent to patients recently diagnosed with cancer. Answers were grouped and compared using Pearson’s chi-square test.

**Results:**

A total of 89.1% of respondents reported having an assigned care provider. These individuals reported higher levels of involvement, information, and emotional support compared to individuals who did not have an assigned care provider. The profession of the care provider had little impact.

**Conclusion:**

An assigned care provider is important for maintaining continuity of care. This should be encouraged in cancer care as it leads to better patient experience across all investigated domains. The widely spread use of contact nurses in cancer care in Sweden provides a solid ground for such continuity. The continuous use of patient-reported experience measures promotes more people-centered healthcare practices.

## Introduction

Continuity in care is associated with several benefits for both the healthcare system and patients, such as lesser use of emergency care and reduced mortality [1, 2]. It has also been linked to lower future care needs in an oncology setting [3]. The concept of continuity of care has been defined as informational, relational, and management continuity [4]. Although there are numerous ways to measure continuity of care [5, 6], using patient-reported measures is the best way to characterize patient experience. Contrary to claims-based or register data, patient-reported experience measures (PREMs) assess the patient’s experience of continuity as well as other parameters associated with care [6]. It is also a tool for harnessing the patient’s voice in a structured manner. Understanding patient satisfaction and experiences is a key component in people-centered care as they reflect the needs, values, and preferences of patients and can be used for quality improvement [7, 8]. We have a limited understanding of how access to an assigned care provider (ACP) influences the patient’s experience of involvement, perception of information, and emotional support and have investigated relational continuity by studying these aspects using PREMs.

## Methods

We analyzed data from a Swedish nation-wide survey conducted in patients diagnosed with cancer 6–10 weeks earlier during 2018–2022. The survey covers 28 types of cancer and is described in a previous study [9]. We have analyzed 59,113 responses (response rate 64.6%) to the questions “Were you involved as much as you wanted to in the decisions relating to your care/treatment?”, “Do you feel you have been given sufficient information about the investigation of your illness/state of health?”, and “Did you have the possibility of getting emotional support, if necessary, from the care staff?”. Answers were captured on a Likert scale from 1 (no, not at all) to 5 (yes, completely), grouped as negative (1 and 2), neutral (3), and positive (4 and 5) and compared to whether or not responders reported they had an ACP at the health services, and which profession that care provider had, i.e., a doctor, nurse, or other healthcare personnel. Differences between groups were analyzed with Pearson’s chi-square test using STATA/SE 13.1 [10].

## Results

**Fig. 1:**
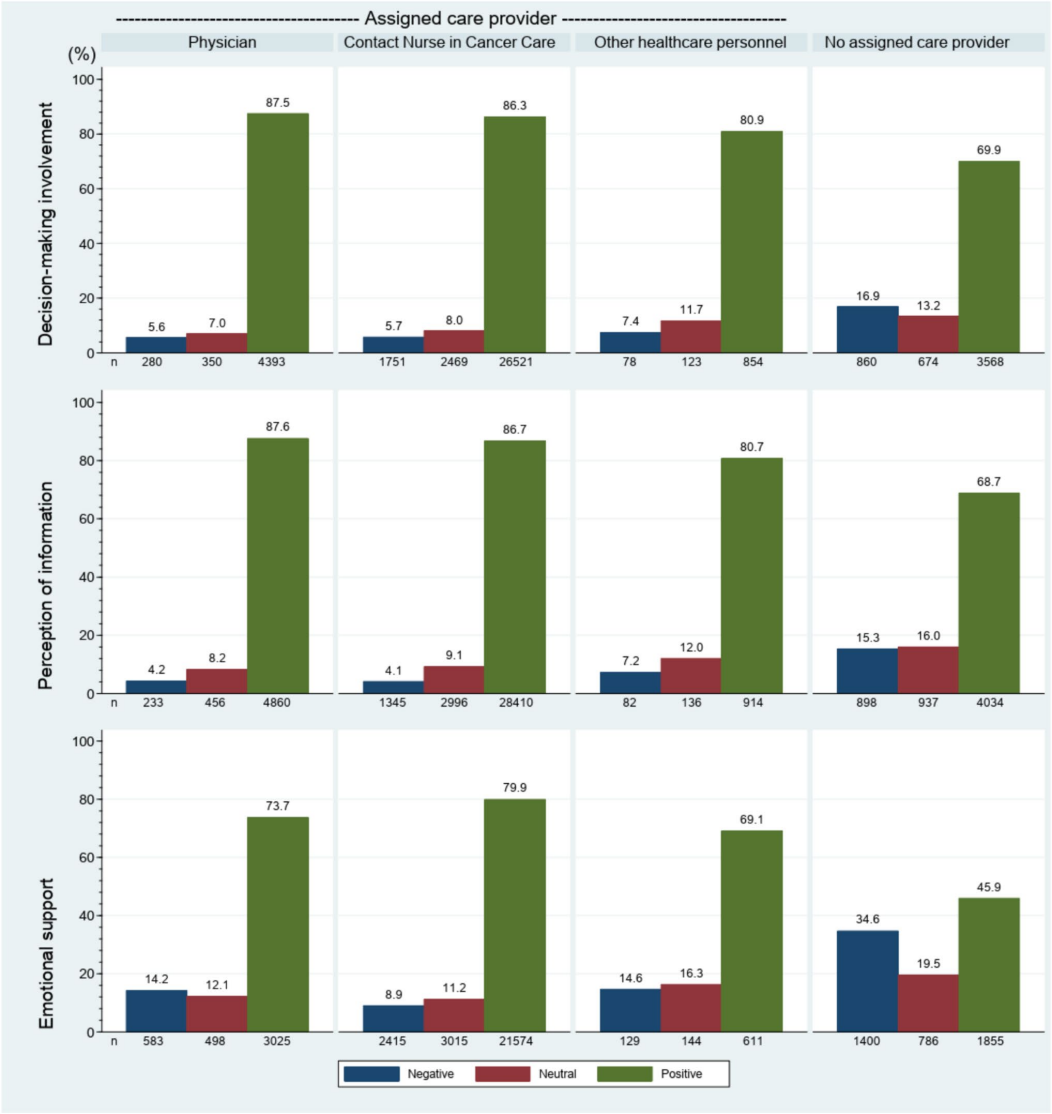
Distribution of ACPs and impact on decision-making, information, and emotional support

An ACP can be described as a care provider who oversees a patient’s care, with responsibility for continuity and coordination. High levels of patient involvement, information, and emotional support were observed in patients who had been assigned a care provider. Physicians, contact nurses, and other healthcare staff as ACP were associated with over 80% positive responses in respect of patient involvement ((87.5% (*n* = 4393), 86.3% (*n* = 26,521), and 80.9% (*n* = 854)) and information ((87.6% (*n* = 4860), 86.7% (*n* = 28,410), and 80.7% (*n* = 914)), whereas patients with no ACP reported only 69.9% (*n* = 3568) and 68.7% (*n* = 4034) positive responses in these areas (*p* < 0.001). Only 45.9% (*n* = 1855) of patients with no ACP were positive to the provided emotional support compared to 69–80% for those with an ACP (*p* < 0.001) (73.7% (*n* = 3025) for physician, 79.9% (*n* = 21,574) for contact nurse, and 69.1% (*n* = 611) for other healthcare personnel) (Fig. 1).

A comparison of the patients’ satisfaction depending on the profession of the ACP showed only small differences between physicians and nurses. The largest difference was shown for emotional support (79.9% vs. 73.7% positive responses, *p* < 0.001) in favor of nurses. Contact nurse is also the most common type of assigned care provider (Fig. 1).

The questionnaire used for this study allowed respondents to give more than one answer to the question regarding ACP. We investigated the impact of having an ACP as well as potential differences between the categories of providers. For this purpose, respondents reporting more than one ACP were excluded from analysis.

## Discussion

Continuous use of PREMs allows for people-centered development of the healthcare system and is a solid base for quality improvement [11]. Relational continuity in the form of an ACP seems to be an important factor affecting involvement, information, and emotional support for patients. It is assumed that meeting the same care provider enables building a personal connection and provides a basis for trust, improved communication, better precision in medical decisions, and better adherence to prescribed treatments [6]. It is reasonable to assume that improved emotional support, information, and involvement share the same pattern.

Evidently, being assigned a care provider is more relevant to patients than the care provider’s profession. Contact nurse in cancer care is an advanced nursing role recently implemented in Sweden [12]. The role of the contact nurse is, among other things, to assess care needs, provide information and support, and establish care plans together with the patient [13]. This holistic approach is in line with international frameworks on people-centered care [11, 14]. Although the set-up differs between hospitals and clinics, a contact nurse is often assigned to the patient at diagnosis and follows the patient during treatment and follow-up [13, 15]. This might be particularly important for patients moving between clinics, meeting several physicians. In our study, a contact nurse in cancer care is by far the most common type of ACP. The regular provider of care is often described as a physician, but our data suggests that a contact nurse in cancer care can provide the same benefits as a physician with respect to patient involvement, information, and emotional support. However, there are likely aspects of information that are better provided to patients either by physicians or nurses showing that the different roles complement each other. In this study, we could only catch the overall perception of information. Results may vary by age, gender, cancer type, or socioeconomic status and these aspects should be investigated further. Contact nurses in cancer care are greatly appreciated by patients [16]. However, the organizational prerequisites differ between hospitals [15] and should be harmonized to provide equal access to relational continuity in cancer care.

Our results demonstrate the importance of ACPs to maintain continuity of care for a better patient experience across all investigated domains. Since physicians and nurses seem to function comparably in this aspect, our results open up novel ways of work force planning, organizing, and delivering people-centered care.

## Data Availability

Data is publicly available in restricted form at https://resultat.patientenkat.se/ (partially in Swedish). Deidentified participant data will be made available from the authors, upon reasonable scientific request and approved ethical board application. Requests for data will be reviewed on a case-by-case basis.
